# Social distancing, emotional suffering, and cognitive performance in mature and older adults: a literature review

**DOI:** 10.1590/1980-5764-DN-2022-0032

**Published:** 2023-05-12

**Authors:** Gabriela dos Santos, Thais Bento Lima-Silva

**Affiliations:** 1 Universidade de São Paulo, Escola de Artes, Ciências e Humanidades, Departamento de Gerontologia, São Paulo SP, Brazil.; 2 Universidade de São Paulo, Faculdade de Medicina, Hospital das Clínicas, Grupo de Neurologia Cognitiva e Comportamental, São Paulo SP, Brazil.

**Keywords:** Physical Distancing, Psychological Distress, Cognition, Aged, Distanciamento Físico, Angústia Psicológica, Cognição, Idoso

## Abstract

Variables related to social distancing can have negative effects on the emotional well-being and cognition of adults and the elderly.

**Objective:**

The aim of this study was to analyze the available studies that address the relationship between situations of social distancing, socioemotional aspects, and cognition in the lives of mature and older adults.

**Methods:**

A literature review study was carried out between December 2021 and January 2022, involving the SciELO, PubMed, and ScienceDirect databases, with studies published between February 2018 and December 2021.

**Results:**

A total of 754 studies were identified, and after selection, 18 were included. Notably, 16 showed significant effects of social distancing on cognition and socioemotional aspects, that is, the greater the social distancing, the lower the capacity for cognitive performance and the higher the index of symptoms of depression and anxiety, for example.

**Conclusions:**

Greater engagement in social activities and a closer contact with friends and family are protective factors against symptoms of depression and anxiety and cognitive decline.

## RESUMO

Variáveis relacionadas ao distanciamento social podem gerar efeitos negativos para o bem-estar emocional e para a cognição de adultos e idosos.

**Objetivo:**

Analisar os estudos disponíveis que abordam a relação entre situações de distanciamento social, sofrimento emocional e cognição na vida de adultos maduros e idosos.

**Métodos:**

Realizou-se um estudo de revisão de literatura entre dezembro de 2021 e janeiro de 2022, que envolveu as bases de dados Scientific Electronic Library Online (SciELO), United States National Library of Medicine (PubMed) e ScienceDirect, com estudos publicados entre fevereiro de 2018 a dezembro de 2021.

**Resultados:**

O total de 754 trabalhos foi identificado e, após a seleção, 18 foram incluídos. Dezesseis mostraram efeitos significativos do distanciamento social na cognição e no sofrimento emocional, ou seja, quanto maior o distanciamento social, menor a capacidade de desempenho cognitivo e maior o índice de sintomas de depressão e ansiedade, por exemplo.

**Conclusões:**

O maior engajamento em atividades sociais e a maior aproximação com amigos e familiares são fatores de proteção contra sintomas de depressão e ansiedade e declínio cognitivo.

## INTRODUCTION

The most recent experience of social distancing was caused by the COVID-19 pandemic. Distancing recommendations (isolation, quarantine, lockdown measures, etc.) were made by health organizations to the population at large, but targeted older individuals in particular owing to this group’s greater number of comorbidities and consequently increased risk of death from the disease^1^. Therefore, besides facing the fear of becoming infected by the disease, older people had to cope with the loss of social interaction, a vital mechanism for leading a normal life.

The nature of population aging today has drawn increasing attention, given the significant impacts of this shift both individually and collectively. Increased longevity and quality of life during the aging process are determined by social, economic, cultural, and environmental factors and are associated with physical and mental health conditions^2–4^.

In recent years, discussions and concerns have increasingly centered on mental health. Evidence shows that one in every three older adults is affected by mental disorders, of which common mental disorder (CMD) is the most prevalent. This disorder is characterized by symptoms such a fatigue, forgetfulness, difficulty in concentration, anxiety, and irritability, although these are not sufficiently intense for a diagnosis of major depressive disorder (MDD) or generalized anxiety disorder (GAD)^5,6^.

Depressive symptoms and depression are often regarded as a natural part of the aging process, but the disease ranks among the leading 10 factors associated with reduced years of life. Depression also constitutes one of the three main determinants of impaired functioning, which can lead to disability in carrying out activities of daily living. Anxiety is diagnosed in 4% of older adults when associated with other mental disorders, such as depression^7,8^.

A significant factor for the development of CMD, MDD, or GAD is social support received through social interactions. Social relationships are connections established between two or more people, such as friends, neighbors, or family members. In the context of situations of assistance between people, these connections are referred to as support networks or social support networks. Where older individuals are concerned, social support networks contribute to good mental and physical health, promoting functional and social capacity gains^9^.

Unlike support, social distancing is harmful for individuals, particularly older people in this case. Distancing can be related to loneliness, which is a situation of perceived subjective loneliness amid a desire to engage with others^10^.

Romero et al. sought to describe the health characteristics, socioeconomic conditions, adherence to social distancing measures, and feelings of sadness or depression in Brazilian older adults during the pandemic period. The results showed that around 50% of the 9,173 respondents experienced sadness or depression. In addition to emotional impacts, a lack of social interaction affects the brain because of a decline in stimuli received^11,12^.

Studies show a strong relationship between social isolation and cognitive decline^13–16^. Read et al., in a study investigating the association between social isolation and memory decline in mature and older adults, found worse memory performance with increasing social isolation^16^.

The decline in intellectual abilities can limit or prevent the performance of routine functions such as speech and language, logical reasoning, and critical thinking, in addition to promoting serious difficulties related to memory^17^. In addition, psychological aspects, such as depression, are also considered risk factors^18^.

The literature reports a significant association between mental health, social distancing, and cognition^19–21^. The objective of the present study was to analyze the available literature addressing the interface between these three aspects in mature and older adults, with an emphasis on recent findings related to social distancing caused as a form of prevention against the aggravations of the COVID-19 pandemic.

## METHODS

An integrative literature review was conducted involving searches of the SciELO (570), PubMed (20), and ScienceDirect (164) databases. The following search terms were employed: (cognição OR cognition) AND (idosos OR elderly) AND (distanciamento social OR social distance) AND (depressão OR depression) AND (ansiedade OR anxiety).

Inclusion criteria were as follows: studies published in English or Portuguese in scientific journals between February 2018 and December 2021, including longitudinal studies; samples comprising adults aged ≥50 years; and assessment and measurement of variables related to social distancing, cognition, and emotional suffering, such as depressive and anxiety symptoms.

The exclusion criteria were as follows: publications of Masters dissertations, doctoral theses, book chapters, letters to the Editor, case studies, systematic reviews or meta-analyses, and research protocols; samples comprising individuals aged <50 years; and articles not reporting at least two of the three variables investigated.

Two independent reviewers followed the Statement of Preferred guidelines Reporting Items for Systematic Reviews and Meta-Analyzes^22^, for the stages of identification, screening, and eligibility of studies. For the initial identification, searches were carried out in the aforementioned databases. In the screening phase, duplicate studies were excluded, and titles and abstracts were analyzed according to the inclusion and exclusion criteria described above. During the eligibility stage, the selected studies were read in full and analyzed considering the same criteria mentioned above. The remaining studies, therefore meeting the inclusion criteria, were included in the integrative review.

**Figure 1:**
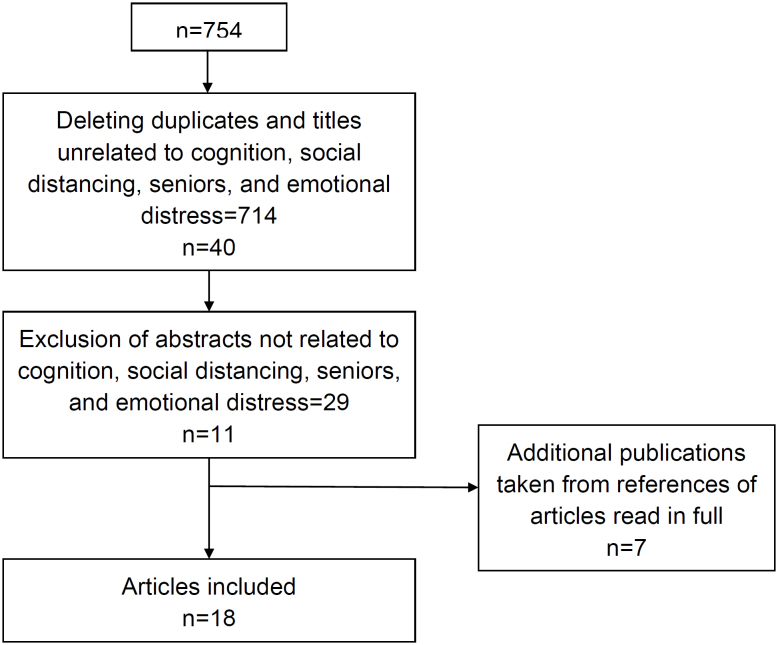
Selection process of articles included in review.

Source: Santos (2022).

A total of 754 records were initially identified. Although not a criterion for inclusion, the studies included were predominantly related to the context caused by the SARS-CoV-2 pandemic; this was possibly due to the search terms used and the period of publication of the chosen studies. Analysis of the studies retrieved was performed in three steps: reading of title, abstract, and full text. After screening of the selected studies, 18 articles addressing cognition, social distancing, older adults, and socioeconomic aspects were included in the review, as depicted in Figure 1.

## RESULTS

With regard to study type, nine^12–14,16,23–27^ were longitudinal, with a maximum time interval of 10 years between the first and last data collections. Regarding sample size, the smallest was a Brazilian study^15^ involving 86 participants, whereas the largest included 11,732 older adults from Japan^28^. Regarding the age groups investigated, 67% of studies evaluated only people aged ≥60 years^12,14,15,24,26,28–34^, whereas the remaining 33% included individuals aged ≥50 years^13,16,23,25,27,35^. Two studies assessed older adults diagnosed with dementia and mild cognitive impairment (MCI) or subjective cognitive decline (SCD), respectively^30,32^.

Data collection took place in different contexts, where six evaluated the scenario caused by the COVID-19 pandemic, a period in which social distancing measures increased worldwide due to the strategy adopted to control the spread of the SARS-CoV-2 virus^15,23,29–32^.

Two studies found weak or no association for the interaction between social distancing and cognitive or emotional suffering^15,23^. Pegorari et al. concluded that older adults with a higher level of loneliness had a greater prevalence of chronic non-communicable diseases^15^. In the study conducted by Besselaar et al., none of the social distancing variables were associated with depressive or anxious symptoms in the participants. According to the authors, this finding might be explained by the greater mastery of coping strategies among older adults^23^.

A synthesis of the articles included in the present literature review is given in Table 1. The descriptions include the authors, publication year, objectives, methods, and results/final considerations of each of the studies reviewed.

**Table 1:** Summary of studies included in review.

Author(s)/year	Objective(s)	Methods	Results/final considerations
Evans et al., 2018^12^	To examine the relationship between social isolation and cognition in later life, and to consider the role of cognitive reserve in this relationship.	Study type: Longitudinal; Sample: Older adults (n=1524); Variables assessed: Sociodemographic, social isolation, cognitive functions, and cognitive reserve.	Social isolation was associated with cognitive function at baseline and 2-year follow-up. Findings suggest that maintaining a socially active lifestyle in later life may enhance cognitive reserve and benefit cognitive function.
Yu et al., 2020^13^	To examine the relationships of social isolation and loneliness on cognitive function among older adult.	Study type: Longitudinal; Sample: Older adults aged ≥50 years (n=7661); Variables assessed: Social isolation, loneliness, and cognitive function.	Loneliness after adjusting for additional confounding variables (chronic diseases, health behaviors, disabilities, and depressive symptoms) was not associated with cognitive decline. By contrast, social isolation was significantly associated with decreases in cognitive functions considering general mental status, particularly episodic memory.
Yang et al., 2020^14^	To investigate the potential mediation mechanism of loneliness on the association between social isolation and cognitive functioning among Chinese older adults within their cultural context.	Study type: Longitudinal; Sample: Older adults (n=7410); Variables assessed: Social activity engagement and cognitive functioning.	Results demonstrated that social activity engagement was significantly related to perceived social isolation. Higher level of social isolation was associated with worse cognitive performance.
Pegorari et al., 2021^15^	To analyze the association of social isolation and loneliness with socioeconomic, clinical, health characteristics, and COVID-19-related variables among older adults.	Study type: Cross-sectional; Sample: Older adults (n=86); Variables assessed: Sociodemographic, psychosocial, physical status, and feelings related to the pandemic.	A moderate positive correlation was identified between loneliness and number of diseases, and a weak positive correlation between loneliness and number of medications and depressive symptoms and risk for sarcopenia. Higher loneliness scores were associated with a greater number of diseases.
Read et al., 2020^16^	To investigate associations between level and changes in social isolation and in memory in older men and women.	Study type: Longitudinal; Sample: Older adults aged ≥50 years (n=11,233); Variables assessed: Aspects of cognitive functioning, socioeconomic variables, social isolation, and depressive symptoms.	Based on date collected between 2002 and 2012, there was a perceived increase in social isolation over time and concomitant decrease in cognitive capacity.
Besselaar et al., 2021^23^	To assess depressive and anxiety symptoms and perceived mastery after the first wave of the COVID-19 pandemic compared to previous years in community-dwelling older adults.	Study type: Longitudinal; Sample: Older adults aged ≥55 years (n=1068); Variables assessed: Depressive symptoms, anxiety symptoms, and functional capacity.	Results revealed no significant increases in depressive or anxious symptoms of participants. This might be because older adults have better coping strategies.
Arai et al., 2021^24^	To investigate the effect of social interaction on BPSD among long-term care facility residents over 1 year.	Study type: Longitudinal; Sample: Older adults diagnosed with dementia/symptoms of pathological cognitive decline (n=220); Variables assessed: Sociodemographic, health conditions, activities of daily living, cognitive functions, and BPSD.	Results showed an association between activity participation, relationships with residents, and communication with family/relatives. Of the outcomes assessed, frequent communication with family/relatives was associated with lower severity of BPSD.
Santini et al., 2020^25^	To analyze the relationship of social distancing, perceptions of social isolation with anxiety, and depression symptoms in older adults.	Study type: Longitudinal; Sample: Older adults aged 57–85 years (n=3005); Variables assessed: Aspects related to social distancing, perceived isolation, and symptoms of depression and anxiety.	Individuals with less social connectedness had higher perceived isolation and higher depression and anxiety symptoms.
Kobayashi and Steptoe, 2018^26^	To examine the associations between social isolation, loneliness, and engagement in health behaviors over 10 years among older adults.	Study type: Longitudinal; Sample: Healthy older adults without dementia (n=3,392); Variables assessed: Social isolation, loneliness, and health behaviors, besides socioeconomic covariables and depressive symptoms.	Individuals with higher levels of social isolation had greater prevalence of depressive symptoms. Loneliness was associated with the presence of chronic diseases.
Lara et al., 2019^27^	To examine the association of loneliness and social isolation on cognition over a 3-year follow-up period in middle- and older-aged adults.	Study type: Longitudinal; Sample: Older adults aged ≥50 years (n=1691); Variables assessed: Loneliness, social isolation, and cognition.	Loneliness and social isolation were significantly associated with lower cognitive scores. The effect of loneliness and social isolation on cognition remained significant after the exclusion of individuals with depression.
Okamoto and Kobayashi, 2021^28^	To assess the relationship between social isolation and cognitive functioning.	Study type: Cross-sectional; Sample: Older adults (n=11,732); Variables assessed: Social isolation index, cognitive functions, health-related aspects, and sociodemographic data.	For both men and women, the association between social isolation and cognitive functioning was significant. This association, however, was not confirmed after further statistical analyses.
Souza-Filho et al., 2021^29^	To identify factors associated with coping with the COVID-19 pandemic by older adults with and without comorbidities.	Study type: Cross-sectional; Sample: Older adults (n=569); Variables assessed: Sociodemographic, occupational activities, and emotions related to COVID-19.	There was a positive association of emotions of the older adults with comorbidities, where this group had twice the odds of reporting frequent crying during the pandemic.
Saraiva et al., 2021^30^	To investigate the relationship between life-space mobility and QoL in older adults with and without frailty during the COVID-19 pandemic.	Study type: Multicenter, prospective cohort; Sample: Older adults (n=557); Variables assessed: Mobility, frailty, demographics, comorbidities, cognition, functionality, QoL, loneliness, depression, and anxiety.	Mobility was restricted in 79% of participants and affected QoL for 77%. No significant results were found for depressive symptoms and anxious symptoms associated with the pandemic were observed in 19% of participants.
Savci et al., 2021^31^	To evaluate the fear of COVID-19, loneliness, resilience, and QoL levels in older adults in a nursing home during the pandemic, and the effects of these variables and descriptive characteristics on QoL.	Study type: Cross-sectional; Sample: Older adult residents of LTCF (n=103); Variables assessed: Sociodemographic, cognition, QoL, resilience, fear, and loneliness.	Results showed that social distancing, loneliness, and physical characteristics had the greatest impact on QoL. Fear was regarded as a protective aspect for health.
Di-Santo et al., 2020^32^	To explore the effects of COVID-19 and quarantine measures on lifestyles and mental health of elderly at increased risk of dementia.	Study type: Cross-sectional; Sample: Elderly without dementia, but diagnosed with MCI or SCD (n=126); Variables a ssessed: Sociodemographic, cognitive functions, functional capacity, factors associated with COVID-19 pandemic, and psychological aspects.	There was an association of reduction in leisure activity with anxiety symptoms. Also, living alone was associated with greater presence of depressive symptoms.
Song et al., 2019^33^	To investigate the significant sociodemographic, lifestyle-related, and disease-related correlates of depressive symptoms among older adults.	Study type: Cross-sectional; Sample: Older adults with MCI (n=154); Variables assessed: Sociodemographic, cognitive functions, and depressive symptoms.	The prevalence of depressive symptoms among Chinese older adults with MCI was 31.8%. The analysis showed that poor perceived positive social interaction, small social network, low level of physical activity, poor functional status, subjective memory complaint, and poor health perception were correlated with depressive symptoms.
Ribeiro et al., 2018^34^	To analyze the relationship between QoL and depressive symptoms of older adults from a domestic setting.	Study type: Cross-sectional; Sample: Older adults (n=228); Variables assessed: Sociodemographic, cognitive functioning, depressive symptoms, and QoL.	The prevalence of depressive symptoms was 31.1%. On the final regression model, the physical, psychological, sensory, and intimacy domains continued to be a protective factor, and social participation was a risk factor for depressive symptoms. Thus, depression symptoms were associated with low perceived QoL in older adults.
Taylor et al., 2018^35^	To investigate the impact of objective and subjective social isolation on depressive symptoms and psychological distress among a national sample of older adults.	Study type: Cross-sectional; Sample: Older adults aged ≥55 years (n=1439); Variables assessed: Socioeconomic, depressive symptoms, objective, and subjective social isolation.	Objective social isolation was unrelated to depressive symptoms, whereas subjective social isolation from both family and friends and from friends only was associated with more depressive symptoms.

Abbreviations: QoL: quality of life; LTCF: long-term care facility; COVID-19: coronavirus disease 2019; BPSD: behavioral and psychological symptoms of dementia.

The importance of social engagement in family relationships, with other residents in the case of long-term care facility (LTCF) residents, and the involvement in leisure activities were considered protective factors against behavioral and psychological symptoms of dementia in elderly patients diagnosed with MCI and anxiety symptoms in healthy older adults^24,32^.

Significant effects of social distancing, or correlated variables, on cognitive functions, depressive symptoms, or anxiety were found in 16 of the studies reviewed^12–14,16,24–35^, although this significance was not confirmed by Okamoto and Kobayashi^28^ following further statistical analyses.

As depicted in Table 2, the main impacts of the variables of social isolation in the studies reviewed were on cognition and depressive and/or anxious symptoms. These results corroborate the arguments of Lewnard and Lo showing that, although the measures promoting social distancing as a strategy to curb viral transmission are necessary, negative outcomes for mental and cognitive health are one of the consequences^36^. The impacts of social distancing are also shown to be harmful to the cognitive abilities of those evaluated in seven studies. As described by Cai^37^, social participation has beneficial effects on cognitive skills; thus, the greater the social interactions, the greater the chances of a good performance in cognition.

**Table 2:** Impacts of variables on social isolation.

Impacts	Reference number
Impact on depression and/or anxiety	29, 30, 25, 26, 32
Impact on cognition	13, 14, 16, 35, 12, 27, 28
Impact on depression, anxiety, and cognition	24, 33
Impact on quality of life	34
Impact on quality of life and depression	31

Due to the fact that a smaller number of studies found impairments for depression, anxiety, and cognition concomitantly, quality of life or quality of life and depression simultaneously refers to the absence of collection of this information in the other studies.

A methodological feature that deserves to be highlighted is the fact that some studies use technological means such as online questionnaires or the use of the telephone for data collection. Despite this being a strategy, even for times that require greater physical restraint between people, conducting data collection for scientific research may present weaknesses, such as those who do not have access to the Internet or a telephone, for example, will not have the opportunity to compose the sample, technical problems or the absence of technological domains can compromise the quality of the answers collected, the questionnaires can receive answers from third parties, and/or that answers do not correspond to the accomplishment of the investigated sample^38^.

The main methodological difference between the longitudinal studies and those that were conducted from the pandemic context refers to the sample size, so studies that were conducted before the pandemic have a smaller number of participants.

Although there are several sociocultural, economic, physical, and mental health factors, among others, in studies that did not have any specific focus or focus on the effects of the pandemic, the motivations and events that influenced social distancing are not clear.

In summary, the results of the analyzed studies indicate that there is an association between emotional distress, with emphasis on depression and anxiety, cognitive performance, and social distancing. The results also demonstrate that the context of the COVID-19 pandemic has been considered when conducting the most recent studies on the subject.

## DISCUSSION

The objective of this literature review was to analyze the available studies addressing the relationship between social distancing, socioemotional aspects, and cognition in the lives of mature and older adults. A total of 18 studies published in Portuguese and English between 2018 and 2021 met the other inclusion criteria and were thus selected for this review.

As presented in the results, 88% of the studies reported a significant positive association between social distancing, socioemotional aspects, especially depression and anxiety, and declines in the cognitive abilities of healthy or MCI-impaired mature adults and older adults. Conversely, two studies found the opposite results, with possible explanations for this disparity put forward by Fontes and Neri^39^.

Due to the biopsychosocial changes promoted by the aging process, the negative effects on well-being and quality of life of older adults tend to be greater^39^. Nevertheless, the older population has more resources to develop abilities and use strategies that can facilitate the process of coping with adversities, such as those posed by the COVID-19 pandemic.

The aim of the study by Garcia-Fernandez et al. was to investigate the emotional state of older Spanish adults during the time of the pandemic, specifically between March and April 2020. A comparison of scores for loneliness, depression, and anxiety in participants aged ≥60 years versus those aged <60 years revealed that the older group had a lower prevalence of depressive symptoms and stress compared to the younger group. In addition, the older individuals also experienced less impact on anxiety, which, according to the authors, was an unexpected phenomenon, given the levels of emotional suffering during the pandemic^40^.

Similar to the findings of Garcia-Fernandez et al., the findings of Daly et al. contradict those of the present review. The authors analyzed the presence of depressive symptoms in the US adult population before and during the period of social distancing. The first data collection took place between 2017 and 2018, whereas the second collection occurred in March and April 2020, i.e., early in the COVID-19 pandemic. According to the authors, the rate of depression increased by 8.7%, except in older individuals and black people, groups showing similar levels at both time points. Similarly, Rohr et al. found that older adults from Germany exhibited no negative impacts on mental health in the initial period of isolation due to COVID-19^40–42^.

In the three cited studies^40–42^, the data were collected at the beginning of the COVID-19 pandemic, as declared by the WHO on March 11, 2020, and this short period investigated may have influenced the outcomes found. This questioning is pertinent since, although older adults have a more resilient profile against adversities, social distancing represents a risk factor for mental and cognitive health, particularly in this population^36,43^.

The systematic review and meta-analysis of John et al. had the aim of investigating the association between affective problems (depression and anxiety) and decline in general cognitive state in older adults, symptoms that, as outlined previously, tend to be more common in situations of lower social participation and engagement. Based on the effect size determined in the 34 studies reviewed, it was concluded that depressive symptoms were associated with a decline in the cognitive abilities of older adults without dementia^44^.

In line with the findings of the present literature review, the results of the systematic review and meta-analysis by Evans et al. found that social isolation is associated with poor levels of cognitive performance in later life. The authors analyzed 51 longitudinal studies by assessing the relationship between social isolation and cognitive function, including global cognitive function, memory, and executive functions. Results suggested that individuals with high engagement in social activity and large social networks had better cognitive function compared with those with low engagement and smaller social networks^45^.

While social isolation impacts different aspects of life, it is mediated by other variables. The integrative review by Bezerra et al. described the main concepts available in the literature published between 2009 and 2019 about the association of social isolation with aging. The authors highlighted three risk factors for this condition: (1) physical and mental health status; (2) sociodemographic characteristics; and (3) the absence of social opportunities^4^.

The first risk factor relates to conditions that impair mobility and human interaction, such as physical disabilities, hearing deficits, dementia, and degenerative diseases. The second factor concerns issues related to gender, social class, education, housing, among others. Individuals who are female, have a low income or educational level, and live in deprived neighborhoods or in LTCF are more prone to be socially isolated. Finally, this final factor is associated with a failure of public policies to favor or encourage social engagement^4^.

Taking this into account, besides external factors, it is important to note the internal factors that favor social self-distancing, the result of disengagement from activities performed with others. Late life can be accompanied by a decrease in social engagement, but unlike the ideas put forward by the Theory of Social Disengagement, this event should not be considered inevitable, because remaining socially engaged, albeit in more restricted activities, can enhance health, quality of life, and well-being^46^.

The evidence gathered showed social isolation to be a risk factor for cognitive health, mental health (most notably increased depressive and anxious symptoms), well-being, sedentarism, development of chronic diseases, and mortality^4,11,47^. Thus, there is a strong cause-effect relationship, often hampering the isolation of the primary factor.

Having a deeper understanding of the factors driving social isolation and its respective consequences can aid in the implementation of preventive strategies. Public policies that foster support networks, promote social engagement, and reduce social inequities can help to prevent social isolation, promote health, and improve quality of life.

An example of public policy that could be adopted as a prevention and protection factor in contexts of less physical-social interaction is the implementation of actions that encourage the digital inclusion of elderly people, since the mastery of certain technologies allows the maintenance of social interaction, even remotely.

The main limitations of this integrative literature review study concern the characteristics of the methods used and, consequently, the identified results. In this sense, the search terms used and the period of publication of the studies considered as inclusion criteria, for example, limited the collection of more diversified studies, including those that presented different results from those found in this review.

This study can contribute to other reviews on the same theme, with studies that seek to investigate the consequences beyond the biological ones caused by social distancing resulting from the COVID-19 pandemic and help in the theoretical basis of actions that aim to promote social interaction as a form of prevention against emotional distress and cognitive decline.

The aging process and late life should be viewed as a whole, whereby physical, mental, and health conditions, along with social, demographic, and economic characteristics, for example, are considered self-influencing factors that determine the characteristics of one another.

In the present literature review, studies investigating the association between social distancing, cognition, and emotional suffering in mature and older adults were analyzed. Taken together, the results provide confirmation that these factors interact and exert a number of effects, including on quality of life.

Future literature reviews on this issue should be conducted involving a broader search of available studies, mapping a timeline, and a comparison of the differences and similarities in methods and results of more recent studies with those published over 5 years ago. This will allow confirmation of whether changes in the characteristics of social distancing have occurred.

Given that the rising number of older adults is an inevitable reality that poses major challenges and opportunities, studies elucidating factors that can have adverse biopsychosocial effects or promote positive benefits for healthy aging and successful old age are crucial for effective planning and management of the life cycle.
